# Global scientific output trend for *Akkermansia muciniphila* research: a bibliometric and scientometric analysis

**DOI:** 10.1186/s12911-020-01312-w

**Published:** 2020-11-10

**Authors:** Hojat Dehghanbanadaki, Hossein Aazami, Shahrbanoo Keshavarz Azizi Raftar, Fatemeh Ashrafian, Hanieh-Sadat Ejtahed, Ehsan Hashemi, Zahra Hoseini Tavassol, Sara Ahmadi Badi, Seyed Davar Siadat

**Affiliations:** 1 Endocrinology and Metabolism Research Center, Endocrinology and Metabolism Clinical Sciences Institute, Tehran University of Medical Sciences, Tehran, Iran; 2 Metabolic Disorders Research Center, Endocrinology and Metabolism Molecular-Cellular Sciences Institute, Tehran University of Medical Sciences, Tehran, Iran; 3 Scientometrics Department, FarIdea Company, Tehran, Iran; 4 Department of Mycobacteriology and Pulmonary Research, Pasteur Institute of Iran, Tehran, Iran; 5 Microbiology Research Center (MRC), Pasteur Institute of Iran, Tehran, Iran; 6 Obesity and Eating Habits Research Center, Endocrinology and Metabolism Clinical Sciences Institute, Tehran University of Medical Sciences, Tehran, Iran; 7 Diabetes Research Center, Endocrinology and Metabolism Clinical Sciences Institute, Tehran University of Medical Sciences, Tehran, Iran; 8 National Research Center for Transgenic Mouse, National Institute of Genetic Engineering and Biotechnology, Tehran, Iran

**Keywords:** *Akkermansia muciniphila*, Microbiota, Bibliometrics, Scientometrics

## Abstract

**Background:**

*Akkermansia muciniphila* is an anaerobic bacterium residing in the healthy intestinal tract of host and its quantity has a negative correlation with various host diseases. This study for the first time provides a holistic bibliometric aspect of *Akkermansi muciniphila* research in the literature and shows the hot topic terms of these articles in any period of time.

**Methods:**

Scopus database was selected to retrieve documents relevant to *Akkermansia muciniphila* in any language up to 2019. The bibliometric profile of *Akkermansia muciniphila* articles including subject area, year distribution, citations, institutions, journals, authors, and countries was systematically characterized and the collaboration networks of authors and countries as well as the burst detection algorithm of the words in the titles, abstracts and keywords were visualized.

**Results:**

There is a progressive growing trend in research on *Akkermansia* from 2004 to 2019 with a total of 566 articles during this period. Out of 353 original articles, there are 194 animal studies (155 studies on mice) and 112 human studies. Also, 65 various diseases were investigated in these studies. The most focused conditions are obesity (71 articles) and type2 diabetes (39 articles). The United States is the leading country on *Akkermansia* publications (n = 132), followed by China (n = 95). Frontiers in Microbiology is the most dominant journal with 23 *Akkermansia* publications. In addition, “cancer” is the hot topic of recent *Akkermansia* research.

**Conclusion:**

*Akkermansia* research is of progressive interest during the last decade and the studies on this subject move towards its relationship with cancer and its promising effect on health.

## Background

Nowadays gut microbiota is considered a significant agent in health maintenance and confronting disease. Recent progress in microbial bionetwork has shown that the balance of the gastrointestinal microbial community is critical to the maintenance of host health [1]. Pro- and prebiotics consumption improves the intestinal flora and plays a definitive role in the prevention or treatment of several diseases [2]. *Akkermansia muciniphila* is one of the beneficial bacteria that considered as a healthy marker in human and introduced as a next-generation probiotic in many diseases. *A. muciniphila* is a Gram-negative, strictly anaerobic, non-motile, mucin degrader bacteria that belongs to Verrucomicrobia phylum as well as constitute 3–5% composition of healthy intestinal microflora [3]. Many researches show that various diseases e.g. obesity, type II diabetes and inflammatory bowel diseases have an association with reducing *A. muciniphila* abundance [4–8]. Due to the close presence of this bacterium to the gut epithelium cells, it has a key role in host-microbiota interaction through regulating host metabolic and inflammatory pathways [5, 9]. Moreover, several studies demonstrated increased intestinal barrier integrity and anti-inflammatory effect of *A. muciniphila* [10–12]. Also, animal and human studies show that *A. muciniphila* consumption could improve several diseases [9, 13, 14]. All these documents reveal the importance of *Akkermansia* on health conditions. Therefore in the current bibliometric study, we are supposed to characterize the recent progress in the research on this domain of literature. Thus, all *Akkermansia* documents based on their article types, year distribution, citations, countries, institutions, authors, funding sponsors, journals, co-authorships, a collaboration between countries, terms and author keywords co-occurrences are systematically analyzed to achieve our goal.

## Methods

In the current study, the Scopus database was selected to retrieve all documents on *Akkermansia muciniphila* due to its wide coverage of literature and high citation records [15]. All documents indexed in Scopus from the beginning up to the end of 2019 were retrieved with no language limitation if their titles, abstracts, and keywords covered the “*Akkermansia muciniphila*” term. The strategy search was as: TITLE-ABS-KEY ("Akkermansiamuciniphila") OR TITLE-ABS-KEY ("A. muciniphila") AND PUBYEAR < 2020. To avoid any changes in citations or other bibliometric indicators, all documents were extracted on the day of searching. Then all data were exported into Microsoft Excel for statistical analysis of the bibliometric information and ranking the prolific data including top-cited documents, top journals, top institutions, top sponsors, top authors, and top countries. We considered all retrieved articles to characterize the different subject areas and document types of *Akkermansia* literature, only original articles to determine the share of each study design, targeted population and conditions/diseases in *Akkermansia* literature and original articles and reviews to assess other bibliometric characteristics including the year distribution of publications, citation numbers, most productive journals, institutions, sponsors, authors, and countries as well as the collaboration between keywords, terms, authors and countries. We utilized Graph Pad Prism (version 8.0.1.244) to display the analyzed data. Following the data extraction, the documents were categorized based on the study design, targeted population and conditions/diseases by two bacteriologists. Also, the VOSviewer (version 1.6.13) (van Eck and Waltman, 2010; www.vosviewer.com), a software tool used to construct, visualize and explore the networks of authors, institutions, countries, keywords, and terms in which the link between the items are based on their co-occurrences, co-authorships, co-citations, bibliographic coupling or citations [16], was applied to map the dense network of the most co-occurrences author keywords and terms as well as the most co-authorships of authors and countries. In addition, the burst detection algorithm of the terms supplying in the titles, abstracts and author keywords of the retrieved articles were visualized through applying Science of Science (Sci2), a software tool used to construct, visualize and explore the temporal map of items supplying in the specific field of science [17]. As the current study is the bibliometric analysis of the published literature, the ethics approval is not required.

## Results

### Publication numbers, subject area, and type of articles

**Fig. 1:**
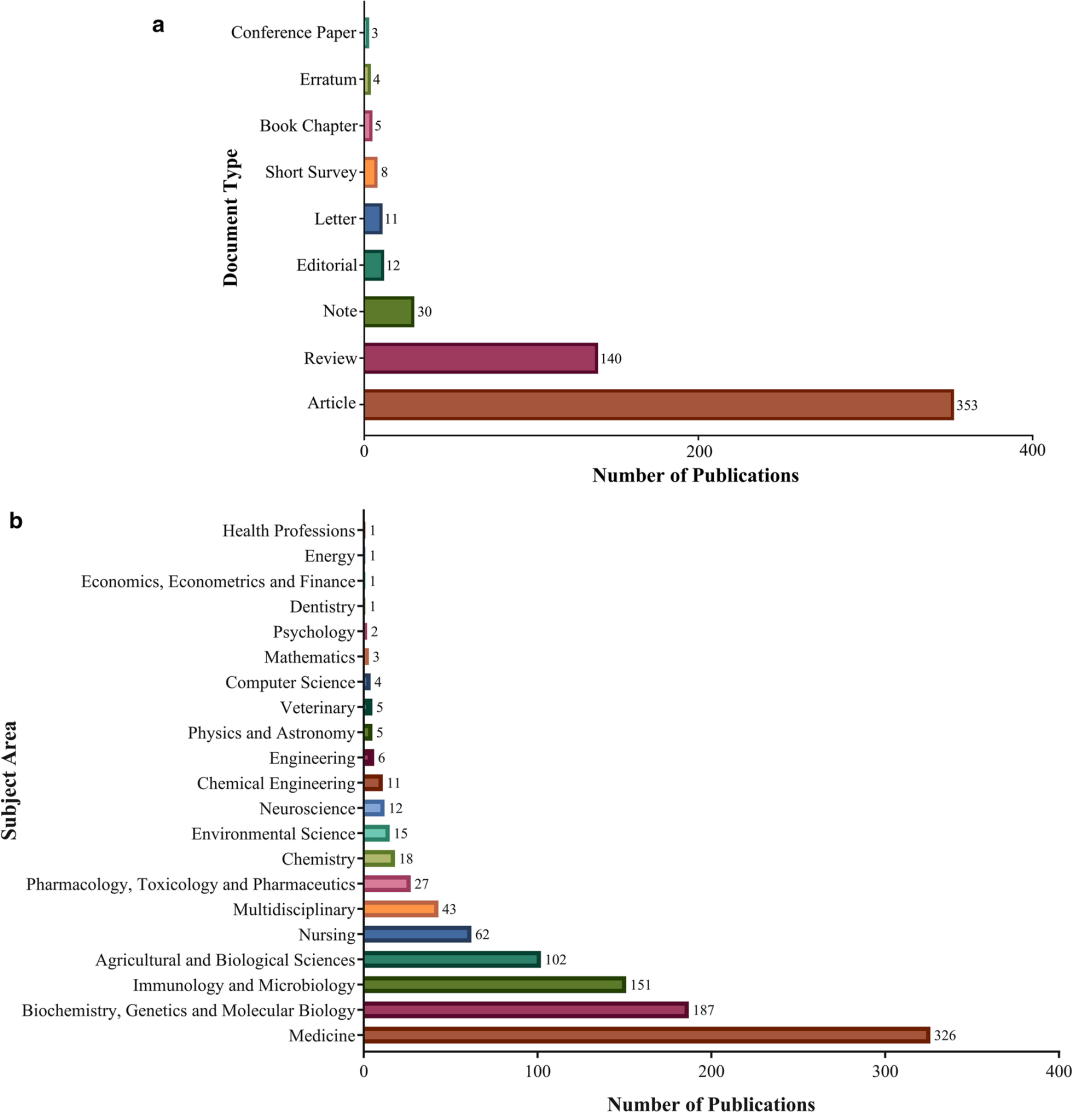
Total articles in *Akkermansia* presents by **a** articles type and **b** subject areas

There are 566 published articles on *Akkermansia* in the Scopus, 311 of which are open accesses and 255 were assigned in others. The great majority of which are original articles (n = 353, 62.3%), followed by 140 review articles (24.7%), 30 notes (5.3%), 12 editorials (2.1%), 11 letters (1.9%), 8 short surveys (1.4%), 5 book chapters (0.9%), 4 errata (0.7%) and 3 conference papers (0.5%) (Fig. 1a). These articles published in four different languages including 556 articles in English, 4 in French, 4 in Chinese and 2 in Russian.

The global *Akkermansia* research output during 2004–2019 has been published in the context of 21 different subfields. 326 documents had published in the area of Medicine that considered as the most focused subject area in this field, followed by Biochemistry, Genetics and Molecular Biology (n = 187), Immunology and Microbiology (n = 151), Agricultural and Biological Sciences (n = 102), Nursing (n = 62), Pharmacology, Toxicology and Pharmaceutics (n = 27), Chemistry (n = 18), Environmental Sciences (n = 15), Neurosciences (n = 12), Chemical engineering (n = 11), Engineering (n = 6), Physic and Astronomy (n = 5), Veterinary (n = 5), Computer sciences (n = 4), Mathematics (n = 3), Psychology (n = 2), Dentistry (n = 1), Economics, Econometrics and Finance (n = 1), Energy (n = 1), Health Professions (n = 1) and 43 documents have multidisciplinay approach (Fig. 1b).

### The distribution of *Akkermansia* articles based on the study design, targeted population and conditions/diseases

We screened the titles and abstracts of all 353 original articles and determined the targeted population of each study and revealed that majority of these studies were conducted in animal population (n = 186 research), followed by human studies (n = 108), in vitro studies (n = 44), in silico studies (n = 6), both animal and in vitro studies (n = 4), both human and animal studies (n = 3), both in vivo and in silico study (n = 1) and one study was conducted in three phases of in vitro, animal and human.

Then, we categorized the type of animals that were utitlized in only animal studies or both animal and other population studies (n = 194) and found that 155 studies were performed in mice, 29 in rat, 2 in poultry and 1 study were performed in each of the following animal population: dog, foals, hamster, marine fish, pig, rabbit and zebrafish.

In addition, we found that *Akkermansia* articles were conducted on 51 various diseases (Additional file 1: Table S1) that the diseases that have attracted the most attention in this regard are as follows: obesity (54 articles), type 2 diabetes (29 articles), inflammatory bowel disease (28 articles) and metabolic disorders (12 articles).

### Year distribution of articles

**Fig. 2:**
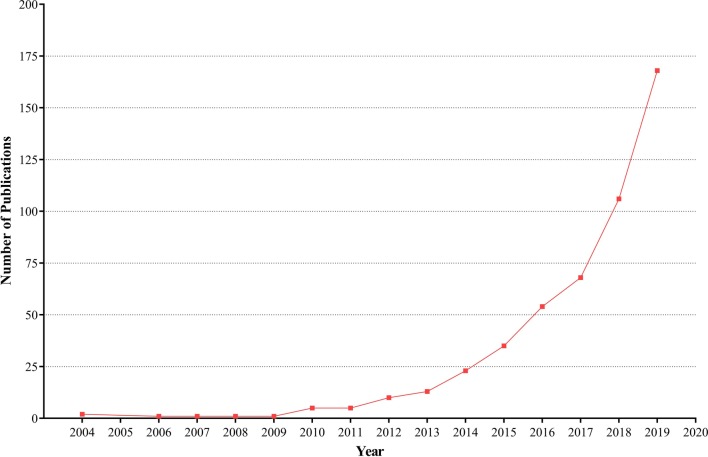
The annual publication in the field of *Akkermansia*. Year distribution of *Akkermansia* articles from 2004 to 2019

The year publication pattern of original articles and reviews on *Akkermansia* is presented in Fig. 2. As shown, the first article indexed in Scopus was published in 2004 in which just 2 articles had been published. After the experience of steady pattern in publication for 9 years, the number of publications in this field had gradually increased and reached 13 articles in 2013 and since then the number of publications remarkably increased and reached top numbers of 168 articles in 2019.

### Citation numbers of published articles and top-cited articles

The total citation number is another main factor for the evaluation of the *Akkermansia* articles in the literature. The lowest citations belonged to the articles in 2004 and the highest was experienced by the articles in 2019 with 6318 citations. The citation number of articles had increased gradually from 2004 to 2014 and then, Annually published articles on *Akkermansia* have received a rapidly growing number of citations (The citations referred to all citations during one specific year for all articles published up to that point). The citation number of each year is depicted in Additional file 2: Fig. S1. In addition, Table 1 demonstrated the characteristics of the 5 top-cited articles on *Akkermansia.* As presented, the top one was published in 2013 in the PNAS journal (Proceedings of the National Academy of Sciences) and cited 1523 times, more information about these articles listed in the table.

**Table 1:** The list of top-cited articles in the field of *Akkermansia*

SCR	First author	Title	Year	Cited by	Source title	Article type	CiteScore (2019)	Access type
1	Everar A	Cross-talk between Akkermansia muciniphila and intestinal epithelium controls diet-induced obesity	2013	1523	Proceedings of the National Academy of Sciences of the United States of America	Original	15.7	Open Access
2	Routy B	Gut microbiome influences efficacy of PD-1-based immunotherapy against epithelial tumors	2018	904	Science	Original	45.3	Open Access
3	Derrie M	Akkermansia municiphila gen. nov., sp. nov., a human intestinal mucin-degrading bacterium	2004	675	International Journal of Systematic and Evolutionary Microbiology	Original	4.2	Open Access
4	Shin N.-R	An increase in the Akkermansia spp. population induced by metformin treatment improves glucose homeostasis in diet-induced obese mice	2014	582	Gut	Original	32.2	subscription
5	Dao M.C	Akkermansia muciniphila and improved metabolic health during a dietary intervention in obesity: Relationship with gut microbiome richness and ecology	2016	528	Gut	Original	32.2	Open Access

### The top active journals in publication

The journals’ contribution (the top-ranked journals) in the field of *Akkermansia* is displayed in Table 2. As seen, the Frontiers in Microbiology, PLoS ONE, Nutrients, Scientific reports and Gut with 23, 17, 16, 15 and 12 articles are the top-ranked journals in the publication of *Akkermansia* articles. Moreover, the trend of publication of three top-ranked journals is presented in Additional file 3: Fig. S2. Both PLoS ONE and Frontiers in Microbiology journals started to publish articles in the field of *Akkermansia* in 2011 with one publication. Then PLoS ONE sharply increased its publications in this field to reach a peak in 2013 with 4 articles. Then it experienced some fluctuation to reached 2 publications in 2018. Meanwhile, Frontiers in Microbiology had published one article per each year of 2014 and 2015 and then started to rise up and reached a hike in 2017 with 7 articles. Besides, the juvenile and member of the top-ranked journal in this field is Nutrients which started to publish in 2015 (just five years ago) with 1 article. Then it has a growing trend and increasingly raise the number of its publication each year and reached 12 publications in 2019.

**Table 2:** Journals contributions in the field of *Akkermansia*

SCR	Source title	Number of publications
1	Frontiers in Microbiology	23
2	Plos One	17
3	Nutrients	16
4	Scientific Reports	15
5	Gut	12

### The profile of top-ranked countries

The number of publications of the top 5 countries is as follows: the USA with 132 articles is the leading country publishing articles on this domain, followed by China with 95 articles, The Netherland with 54 articles, Finland with 47 articles and France with 34 articles (Table 3).

**Table 3:** The profile of top-ranked countries in the publication of *Akkermansia* articles

SCR	Country	Number of publications
1	United States	132
2	China	95
3	Netherlands	54
4	Finland	47
5	France	34

### Top productive institutions in the publication

The most productive institutes based on the number of *Akkermansia* publications are listed in Table 4. Wageningen University and Research Centre was nominated as the most productive and active institution in the publication of *Akkermansia* articles as it has supported 43 documents in this field. The other top productive institutes are Helsingin Yliopisto, Universite Catholique de Louvain, University of Copenhagen and Inserm with 36, 20, 18 and 16 articles, respectively.

**Table 4:** Top productive institutions in the publication of *Akkermansia* articles

SCR	Affiliation	Number of publications	Country
1	Wageningen University and Research	43	Netherlands
2	Helsingin Yliopisto	36	Finland
3	Université Catholique de Louvain	20	Belgium
4	Københavns Universitet	18	Denmark
5	Inserm	16	France

### Funding sponsors

The most active sponsors for *Akkermansia* research are as follows: the National Natural Science Foundation of China, the National Institutes of Health in the USA, European Research Council in Belgium, European Commission in Belgium and Netherlands Organisation for Scientific Research with 49, 49, 32, 10 and 9 articles, respectively (Additional file 4: Table S2).

### Profile of the most active authors

The most active authors are as follows: De Vos, W.M. with 41 publications is ranked as the first, then Belzer C, Cani P.D, Salminen S. and Nielsen D.S with 25, 17, 13 and 11 publications, respectively are the next most active authors publishing *Akkermansia* documents (Table 5). Additionally, the activity of the three top authors during these years is depicted in Additional file 5: Fig. S3.

**Table 5:** List of the most active authors in *Akkermansia* researches

SCR	Author	Number of publications
	De Vos, W.M	41
1	Belzer, C	25
3	Cani, P.D	17
5	Salminen, S	13
	Nielsen, D.S	11

### The most co-occurrences author’s keywords

**Fig. 3:**
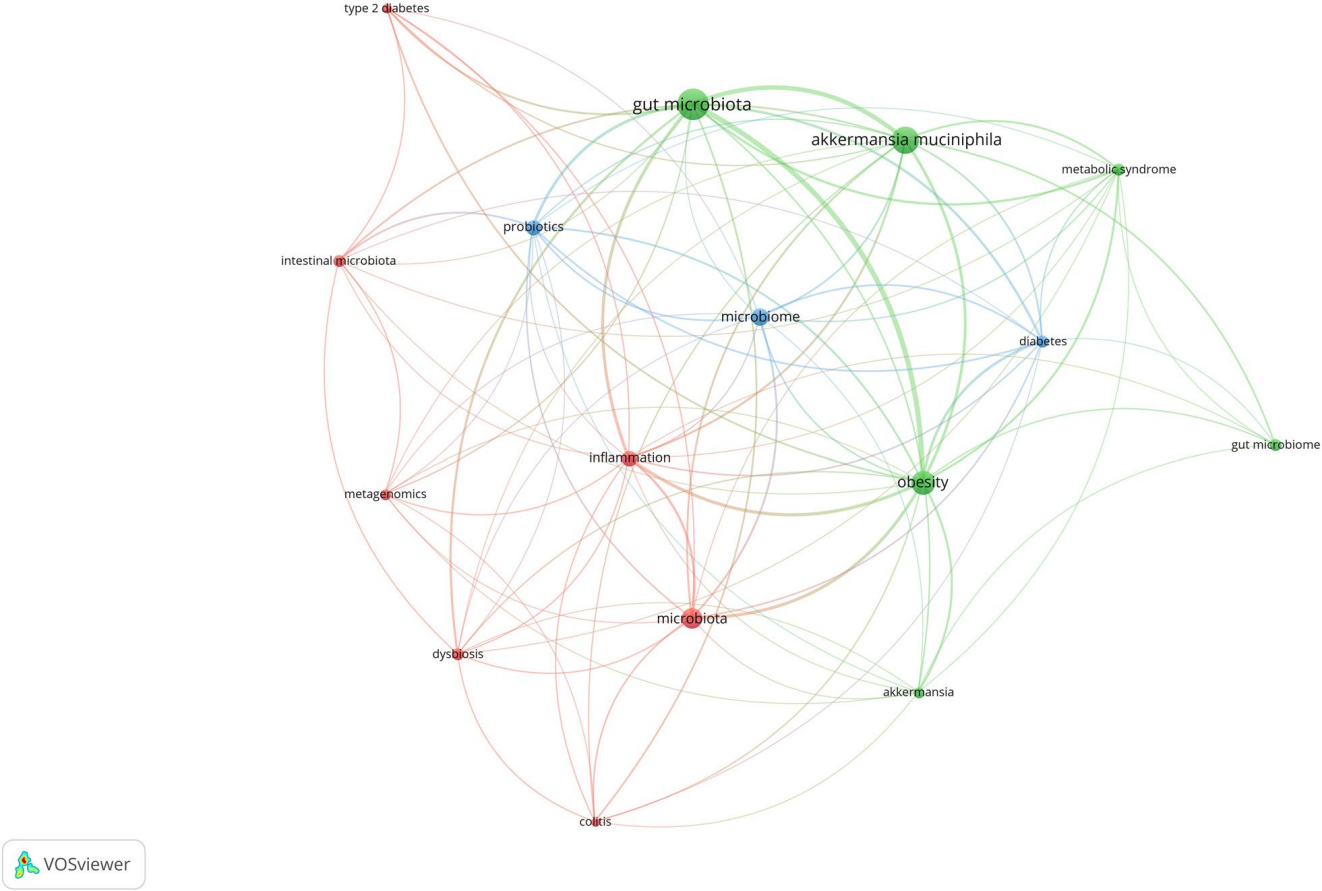
The most frequent author’s keywords. This visualized map of author keywords obtained when minimum occurrences of keywords were imposed at least 10 times. Out of all 967 keywords in this field, 16 meet this threshold that were divided into three clusters showed in different colors. The node size indicates the number of articles that contains that specific keyword

When a criteria of 10 as the minimum number of occurrences for keywords was imposed, 16 of all 967 keywords meet this threshold that include “gut microbiota” (n = 110 co-occurrences), “*Akkermansia muciniphila”* (n = 80), obesity (n = 62), microbiota (n = 48), microbiome (n = 36), probiotics (n = 27), inflammation (n = 26), “intestinal microbiota” (n = 18), “metabolic syndrome” (n = 17), dysbiosis (n = 17) and “gut microbiome” (n = 17). From these keywords, it is clearly can be seen that the most co-occurrences keywords of authors are more related to gut microbiota and its relevant terms (Fig. 3).

### The most co-occurrences terms

**Fig. 4:**
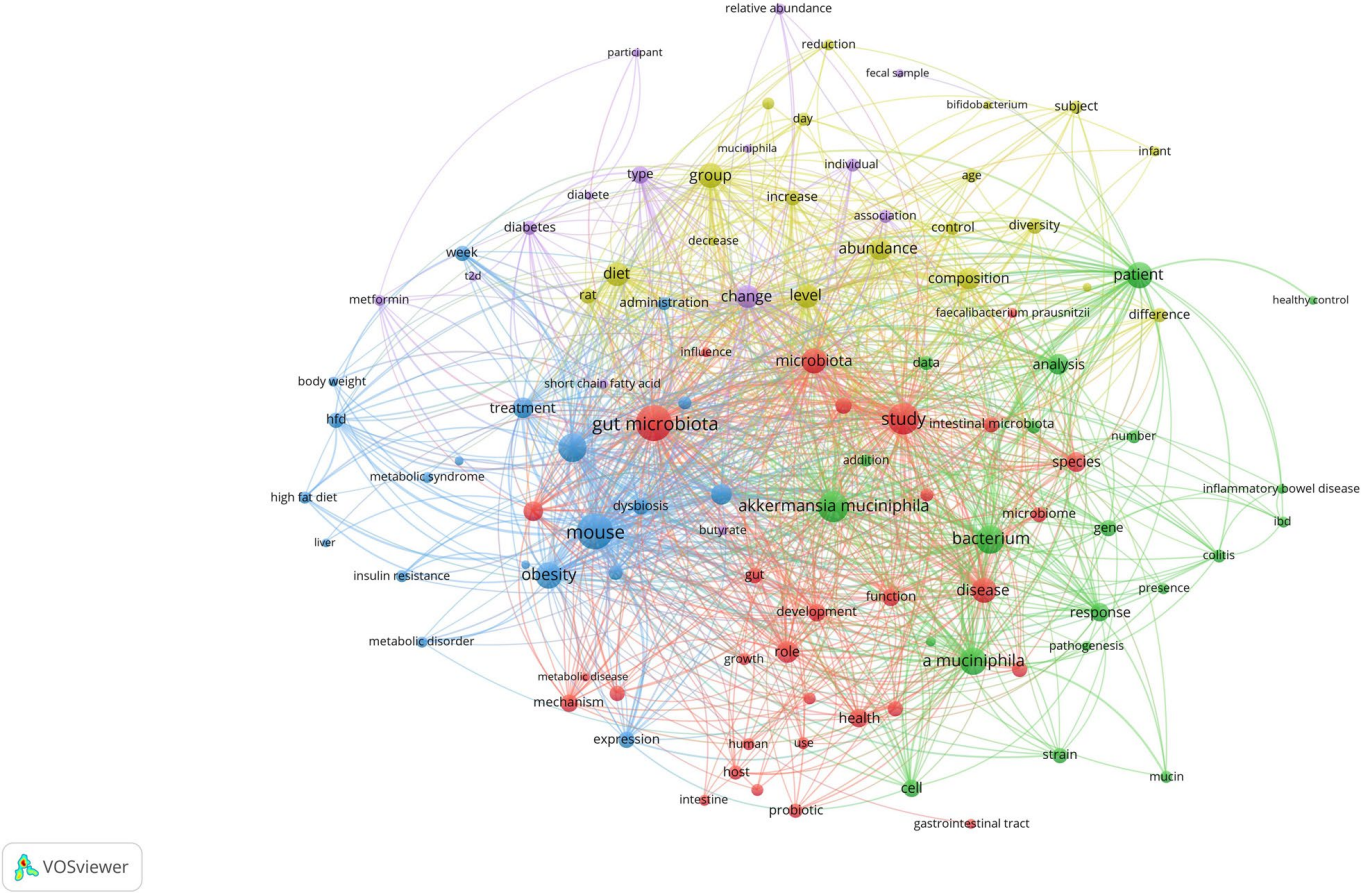
Visualized cluster map of frequent terms. The minimum occurrences for each term were 30, which 105 ones meet the threshold. Then, all included terms were divided into five clusters showed in different colors. The node size of each term indicates the number of articles that contains that specific term

The most co-occurrences terms in the *Akkermansia* articles were Mouse (n = 586 co-occurrences), “Gut microbiota” (n = 576), Study (n = 438), “*Akkermansia muciniphila*” (n = 416), Effect (n = 362), Bacterium (n = 359), “*A. muciniphila*” (n = 332), Obesity (n = 322), Patient (n = 306), Microbiota (n = 298) and Disease (n = 293). These terms were achieved when a minimum number of occurrences for a term was imposed as 30. Thereafter from the 12,129 terms, 105 meet the threshold. These co-occurrences terms were categorized into 5 different clusters that is demonstrated in Fig. 4.

### Co-authorship of authors and countries

**Fig. 5:**
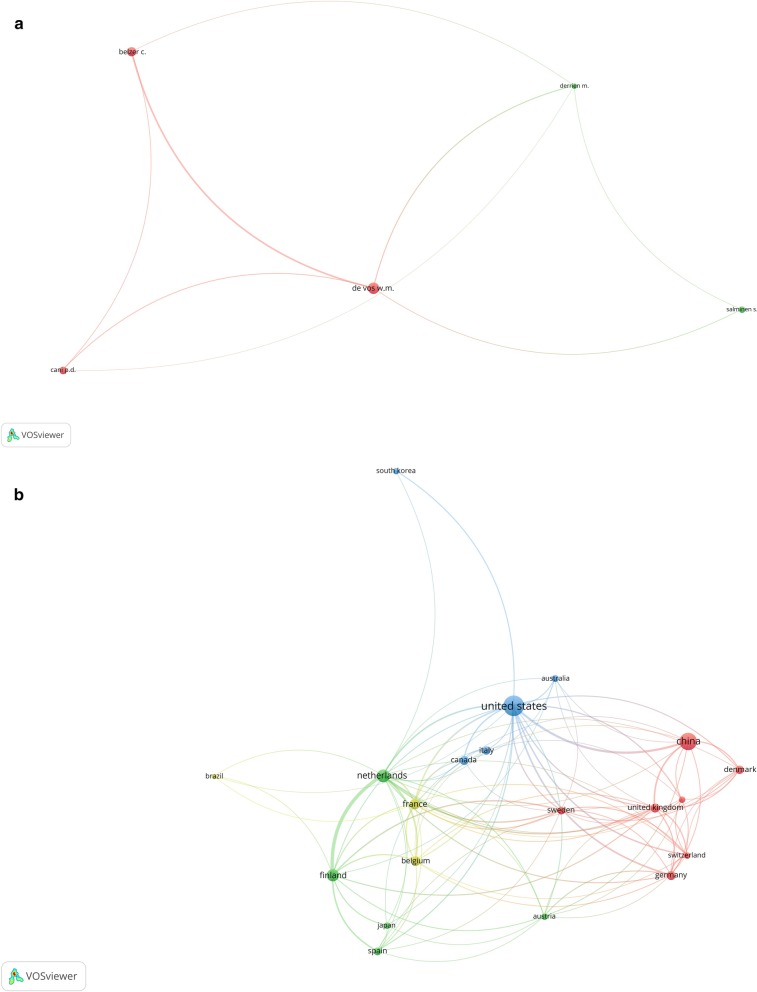
The visualized network of the Co-authorship between **a** authors and **b** countries in the field of *Akkermansia*

The author co-authorship in the publication of *Akkermansia* is displayed in Fig. 5a. To exclude some co-authorships, the minimum number of documents of an author is selected as 10 documents and from 2891 authors, 6 meet the threshold that one of them were excluded due to no co-authorship with other 5 authors. Furthermore, the countries partnership in the publication of *Akkermansia* is displayed in Fig. 5b. To achieve such results, the numbers of collaborative published articles were analyzed and 10 documents were selected as a minimum number of documents of a country and from 61 countries, 20 of which met the threshold. Moreover, the collaborations between 20 countries resulted in 100 contributions (Fig. 5b).

### High frequent burst words in titles, abstracts, and author keywords

**Fig. 6:**
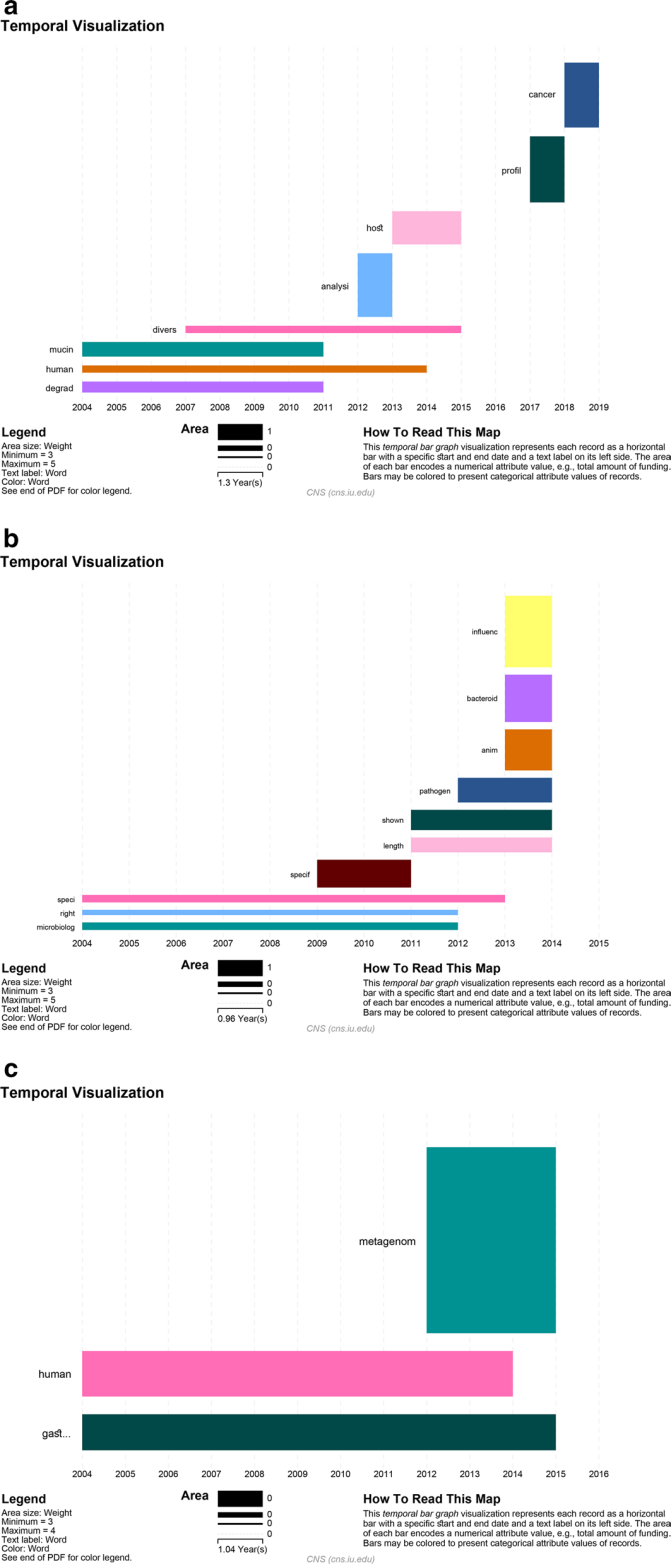
The temporal bar graph visualization of the top bursting terms supplying in **a** the titles, **b** the abstracts and **c** the author keywords of *Akkermansia* articles. The horizontal axis reperesents the year in which each term had emerged and faded. The area size of each bar graph represents the weight of the terms which indicates both the length of time the term was a hot topic and the frequency of the term during this period

As can be seen in the temporal bar graph of Fig. 6a, based on the burst detection of the words in titles of *Akkermansia* documents, there were 8 bursting words in this domain. The top bursting words from the beginning to the end can be categorized into two periods. In the first period, from the beginning (2004) to 2011, there were 4 top bursting words including “degrade” (2004–2011), “human” (2004–2014), “mucin” (2004–2011) and “divers” (2007–2015) that among these bursting words, the weight of mucin is more than other words that indicate the word of “mucin” had been appeared in the titles of *Akkermansia* articles more than other words based on the duration of its usage and its frequency. In the second period, 2011–2019, “analysis”, “host”, “profile” and “cancer” were the top bursting words that weighted 3.21, 3.34, 3.32, and 3.26, respectively. This analysis revealed that the latest works focused on profiling, analysis methods and the relation between *Akkermansia* and cancer. Regarding bursting words in abstract, visualize analyzing of abstracts extracted 10 bursting words, which most of them were between 2009 and 2014. After 2014 there were no bursting words. “Speci”, started from 2004 to 2013, was the longest bursting word in the abstracts and based on the weight, the “influence” had the highest weight of 4.87 (Fig. 6b). In the author's keywords analysis, “gastrointestine” had been burst for a long period of time; from the beginning (2004) to 2015. Also, the latest bursting word was “metagenome” which has the highest weight of 3.99. By introducing advanced high throughput techniques like next-generation sequencing (NGS), a new line of research has been raised and metagenome is one of that approaches which by studying genetic materials presents a large amount of information for researchers (Fig. 6c). The weight of top bursting words in the titles, abstracts and the author keywords of *Akkermansia* articles with their starting and ending time were summarized in Table 6.

**Table 6:** The top bursting words supplying in the titles, abstracts and the author keywords of *Akkermansia* articles

Word	Weight	Start	End
*Title*			
analysi	3.214735137	2012	2013
cancer	3.267532115	2018	2018
mucin	5.164036955	2004	2011
human	3.611350165	2004	2014
host	3.34248467	2013	2015
profil	3.328802011	2017	2018
degrad	3.856778086	2004	2011
divers	2.930580854	2007	2015
*Abstract*			
anim	2.799461206	2013	2014
shown	4.150368781	2011	2014
right	2.857845707	2004	2012
length	3.034339555	2011	2014
influenc	4.879676306	2013	2014
speci	4.601151137	2004	2013
pathogen	3.354185261	2012	2014
microbiolog	4.112075665	2004	2012
bacteroid	3.226980795	2013	2013
specif	3.782564	2009	2011
*Author keywords*			
gastrointestin	2.831938749	2004	2015
human	3.256535242	2004	2014
metagenom	3.996192455	2012	2015

### Discussion

The main aim of this study was to evaluate the scientific trends in *Akkermansia* publications from the bibliometric point of view. The published documents were evaluated from the first report in the field (2004) to 2019. These data revealed that *Akkermansia* is a new trend in recent research and during these two decades have been continuously risen to reach a peak in 2019. By introducing new advanced tools, a new line of research has been opened up and scientists try to realize the relationship between microbiome composition and health conditions.

Gut microbiota composition is associated with various diseases like obesity, inflammatory bowel diseases and type II diabetes. Changes in intestinal microbiota composition in diseases were characterized by increased Proteobacteria phyla and decreased anti-inflammatory bacteria, such as *Akkermansia muciniphila* [18–20]*.* The mucosa-associated microbiota consists of a diverse population that are influenced by the close vicinity of the epithelial layer and nutrients [21]. One of the central players in this population is *Akkermansia muciniphila*. The mucin-degrading bacteria which has been shown to have important consequences on microbiome composition and host physiology [22]. Multiple dietary interventions research has been reported to influence *Akkermansia* spp. abundance in the intestine. In animal studies, the administration of any kind of nutrients that increased the amount of *Akkermansia muciniphila* improved host physiological conditions consequently [23–28].

Based on our results, the importance of this field is the interest of some developed countries like the USA, China, Netherland, Finland, and France. The pattern for most productive institutes was also like top countries as the American and Chinese, Belgian and Finnish institutes were the most productive institutes. Advanced countries like USA always pay more attention to their health care system and try to apply new and high-tech technology in their health strategies and even for their financial purposes. As gut microbial community is a significant agent in health maintenance the gastrointestinal tract of host, and the recent studies have shown that the correlation of gut health and gut composition with most of non-communicable diseases, this can be conclude the more attention to the health of gut, the more human community you will have. The most frequent author's keywords are gut microbiota, *Akkermansia muciniphila*, microbiota and microbiome which are all related to the intestinal microbial composition and discovered the importance of the microbial composition of the intestine in some diseases and recently it was of interest and leads to risen of a new line of science named as Microbiota. In addition, Mouse, Gut microbiota, Study, *Akkermansia muciniphila,* Effect, Bacterium, *A. muciniphila*, Obesity, Patient, Microbiota and Disease are the most frequent terms and also in another part, we showed 194 studies were conducted on animal population while 112 studies were conducted on human population that these data suggest that animal studies formed a large part of current research on *Akkermansia* and maybe it is time to systematically investigate the animal and human studies and evaluate the pooled effect of *Akkermansia* on various diseases and also discover the area of literature in which the results of animal or human studies are inadequate and therefore moved toward filling in the gaps of the science. The USA as pioneer of Akkermansia researches; owing to important of gut microbiome composition, try to identify the microbime composition of its community and utilized this new and advanced knowledge in decision making of health strategies and in the near future personalized medication based on the human gut microbiome.

## Limitation and strenght

To our knowledge, this study is the first detailed bibliometric analysis on the *Akkermansia muciniphila* publications as well as the first descriptive evaluation for the study design, targeted population and conditions/diseases investigatd in this area of literature. There are several databases for data retrieval of this kind of study such as Scopus, Web of Science and Google Scholar and we selected the Scopus due to its wide coverage of literature and high citation records [15]. But our study only limited to Scopus-indexed articles and the other relevant articles of non-Scopus journals were missed though they might contributed to bibliometric characteristics.

### Conclusion

The current study evaluates various bibliometric aspects of all *Akkermansia* articles indexed in the Scopus and gives a complete perspective of the direction of research in this domain over time that it is revealed that *Akkermansia* research is of progressive interest during the last decade. Also, with respect to burst detection of the terms appearing in the titles, abstracts and keywords of these articles, the emerging and fading of hotspot words with their frequency gives the main concept of these articles in each period of time. Accordingly, the burst detection analysis shows the studies on this subject move towards the relationship of *Akkermansia* with cancer and its promising effect on health. We also suggest that the future mainstream research on this domain will be on the association of *Akkermansia* and various health-related conditions of the host and its preventive and therapeutic effects.

## Supplementary information

**Additional file 1: Table S1.** The list of all diseases that have already been evaluated in the *Akkermansia* articles.

**Additional file 2: Figure S1.** The yearly citation numbers of published articles in the field of *Akkermansia*.

**Additional file 3: Figure S2.** The top active journals in publication. The list and number of publications published by journals in the field of *Akkermansia*.

**Additional file 4: Table S2.** The list of top publication funding sponsors in the *Akkermansia* field.

**Additional file 5: Figure S3.** The profile of active authors in the field of *Akkermansia*; author name and annual publication numbers.

## Data Availability

Not applicable.

## Abbreviations

*A. muciniphila*: *Akkermansia muciniphila*
VOSviewer: Visualizing scientific landscapes software
Sci2: Science of Science
PNAS: Proceedings of the National Academy of Sciences
USA: The United States of America
