# Supplementary material for: Global scientific output trend for Akkermansia muciniphila research: a bibliometric and scientometric analysis
Source: BMC Med Inform Decis Mak. 2020 Nov 10;20:291. doi: 10.1186/s12911-020-01312-w (PMC7654583; doi:10.1186/s12911-020-01312-w)
Supplement: Supplementary file 1 — Additional file 1: Table S1. The list of all diseases that have already been evaluated in the Akkermansia articles. [file 12911_2020_1312_MOESM1_ESM.docx]

Supplementary Table S1. The list of all diseases that have already been evaluated in the *Akkermansia* articles.

| **Disease** | **Number of Study** |
| --- | --- |
| Obesity | 54 |
| Type 2 diabetes | 29 |
| Inflammatory bowel disease (IBD) | 28 |
| Metabolic disorders | 12 |
| Ageing | 7 |
| Hyperlipidaemic | 5 |
| Irritable bowel syndrome (IBS) | 5 |
| Multiple sclerosis (MS) | 5 |
| Nonalcoholic fatty liver disease (NAFLD) | 5 |
| Atopic dermatitis | 4 |
| Spondyloarthritis | 3 |
| Autism | 3 |
| Allergic asthma | 2 |
| Chronic kidney disease (CKD) | 2 |
| Hypertension | 2 |
| Alcoholic liver disease | 2 |
| Colorectal cancer (CRC) | 2 |
| Parkinson's disease (PD) | 2 |
| Type 1 diabetes | 2 |
| Hepatocellular carcinoma (HCC) | 1 |
| Rice allergy | 1 |
| spaceflight-related metabolic disorder | 1 |
| Acute appendicitis | 1 |
| Alzheimer | 1 |
| Amyotrophic lateral sclerosis (ALS) | 1 |
| Arthritis | 1 |
| Atherosclerosis | 1 |
| Cedar pollinosis patients | 1 |
| Cryptosporidium infection | 1 |
| Depression | 1 |
| Epithelial tumors | 1 |
| Esophageal adenocarcinoma | 1 |
| Gastrointestinal discomfort and constipation | 1 |
| Gestational malaria | 1 |
| Gut Inflammation in Salmonella Typhimurium-Infected | 1 |
| HIV | 1 |
| Leaky gut syndrome | 1 |
| Multiple system atrophy (MSA) | 1 |
| Niemann-Pick disease (NPC) | 1 |
| Pancreatic cancer | 1 |
| Pancreatitis | 1 |
| Periodontitis | 1 |
| Phenylketonuria (PKU) | 1 |
| Primary sclerosing cholangitis | 1 |
| Progeria (HGPS) | 1 |
| Prostate cancer | 1 |
| Psoriasis | 1 |
| Stroke | 1 |
| Symptomatic uncomplicated diverticular disease (SUDD) | 1 |
| Undernourished | 1 |
| Urticaria | 1 |
