# Supplementary figures and images for: Global scientific output trend for Akkermansia muciniphila research: a bibliometric and scientometric analysis
Source: BMC Med Inform Decis Mak. 2020 Nov 10;20:291. doi: 10.1186/s12911-020-01312-w (PMC7654583; doi:10.1186/s12911-020-01312-w)

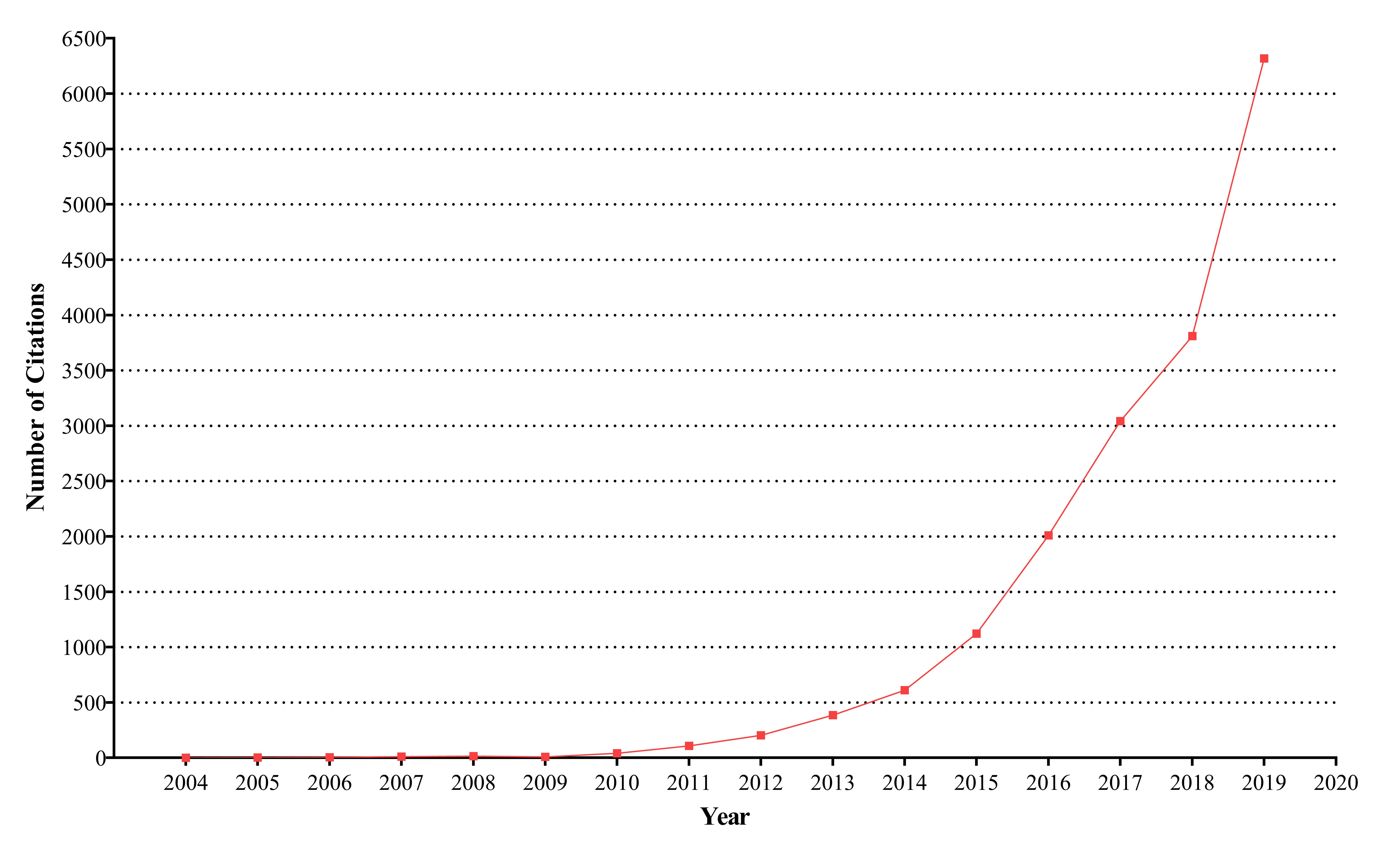

Supplement: Supplementary file 2 — Additional file 2: Figure S1. The yearly citation numbers of published articles in the field of Akkermansia. [file 12911_2020_1312_MOESM2_ESM.jpg]

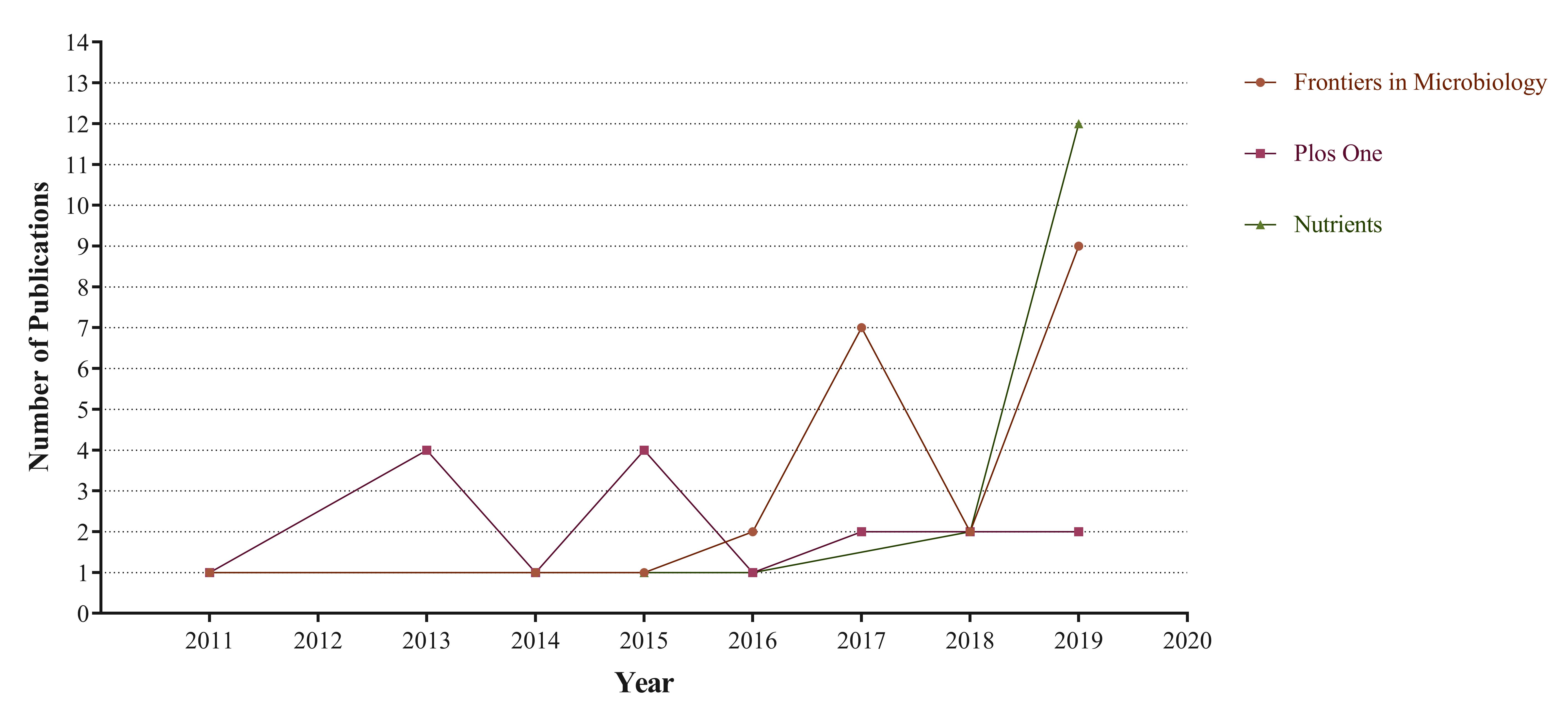

Supplement: Supplementary file 3 — Additional file 3: Figure S2. The top active journals in publication. The list and number of publications published by journals in the field of Akkermansia. [file 12911_2020_1312_MOESM3_ESM.jpg]

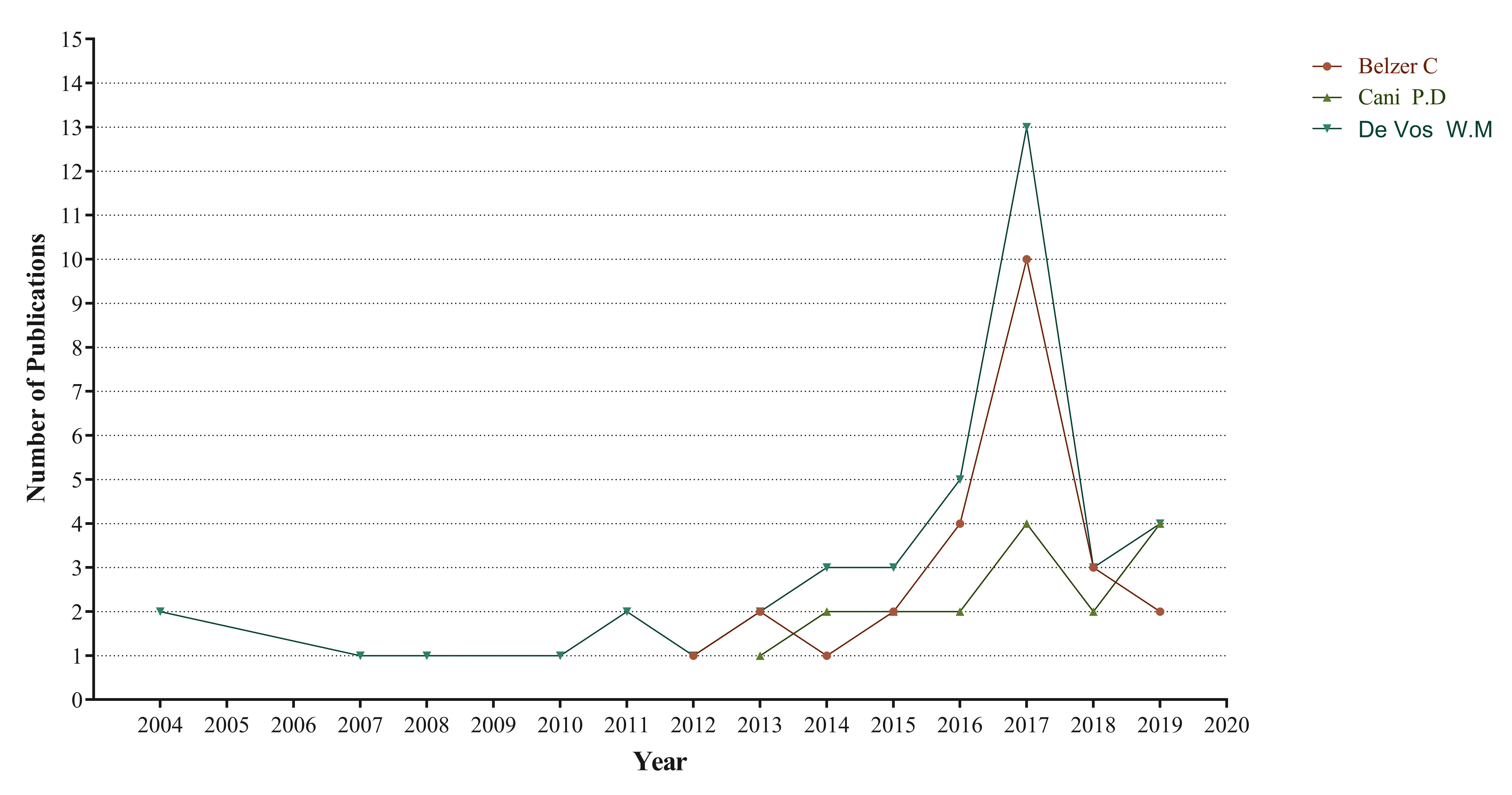

Supplement: Supplementary file 5 — Additional file 5: Figure S3. The profile of active authors in the field of Akkermansia; author name and annual publication numbers. [file 12911_2020_1312_MOESM5_ESM.jpg]
