# Supplementary material for: Global scientific output trend for Akkermansia muciniphila research: a bibliometric and scientometric analysis
Source: BMC Med Inform Decis Mak. 2020 Nov 10;20:291. doi: 10.1186/s12911-020-01312-w (PMC7654583; doi:10.1186/s12911-020-01312-w)
Supplement: Supplementary file 4 — Additional file 4: Table S2. The list of top publication funding sponsors in the Akkermansia field. [file 12911_2020_1312_MOESM4_ESM.docx]

| No | Funding Sponsor | Number of Publications | Country |
| --- | --- | --- | --- |
| 1 | National Institutes of Health | 49 | United States |
| 1 | National Natural Science Foundation of China | 49 | China |
| 3 | European Research Council | 32 | Belgium |
| 4 | European Commission | 10 | Belgium |
| 5 | Nederlandse Organisatie voor Wetenschappelijk Onderzoek | 9 | Netherlands |

Supplementary Table S2. The list of top publication funding sponsors in the *Akkermansia* field.
